# Supplementary material for: Reduction Effect of Carbon Emission Trading Policy in Decreasing PM2.5 Concentrations in China
Source: Int J Environ Res Public Health. 2022 Dec 3;19(23):16208. doi: 10.3390/ijerph192316208 (PMC9738372; doi:10.3390/ijerph192316208)
Supplement: Supplementary file 1 [file ijerph-19-16208-s001.zip › ijerph-1964353-supplementary.pdf]

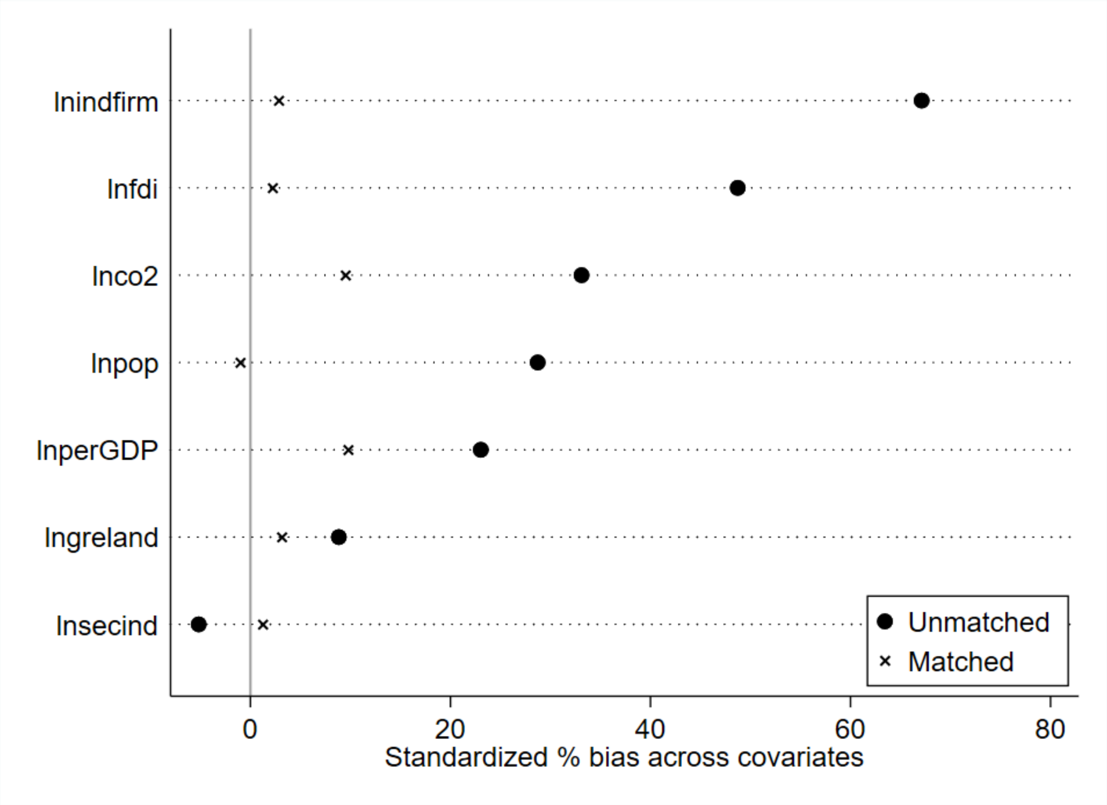

Figure S1. Standardized % bias across covariates between unmatched and matched observations.

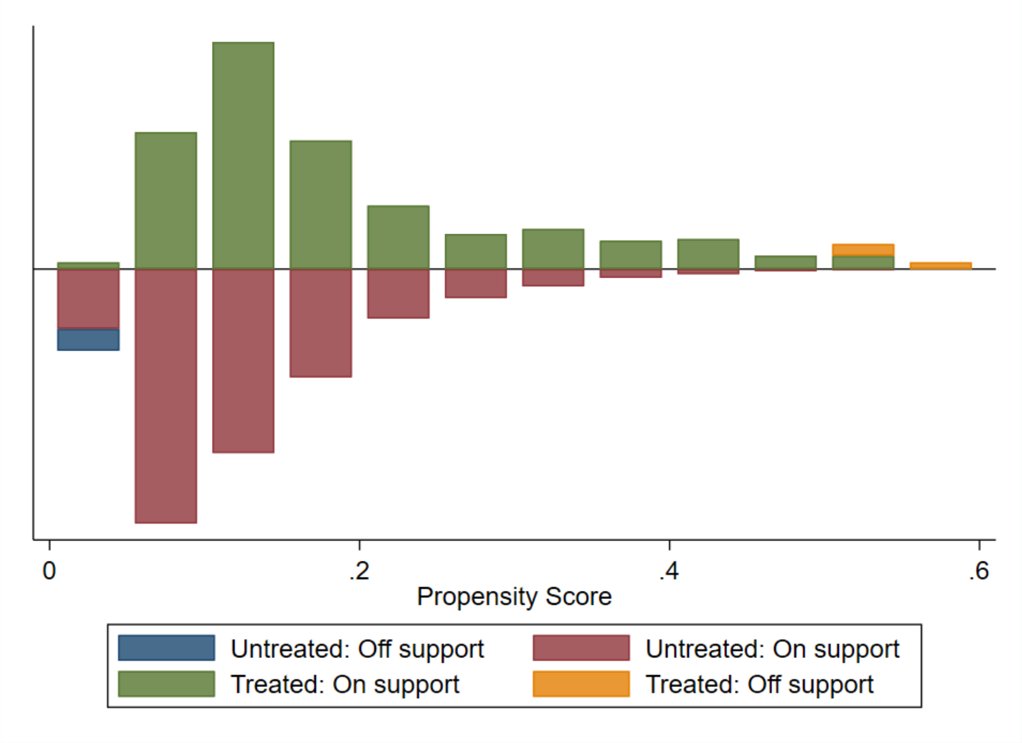

Figure S2. Common support between treated and untreated observations.

Table S1. Robustness of the effects of carbon trading in reducing $PM_{2.5}$ concentrations.

Variable	$lnPM_{2.5}$ (1)	$PM_{2.5}$ (winsor) (2)
ctspost	−0.0950***	−3.1***
	(0.0143)	(0.6)
Controls	YES	YES
City fixed-effect	YES	YES
Year fixed-effect	YES	YES
N	3,258	3,400
Adjusted R square	0.9451	0.9336

Notes: Standard errors are clustered at the city level with robust standard errors in parentheses. "N" represents the number of observations. Significance levels: ***p<0.01.
